# Impact of supine versus upright exercise on muscle deoxygenation heterogeneity during ramp incremental cycling is site specific

**DOI:** 10.1007/s00421-021-04607-6

**Published:** 2021-02-11

**Authors:** Richie P. Goulding, Dai Okushima, Yoshiyuki Fukuoka, Simon Marwood, Narihiko Kondo, David C. Poole, Thomas J. Barstow, Shunsaku Koga

**Affiliations:** 1 Laboratory for Myology, Vrije Universiteit, O|2 Labgebouw, De Boelelaan 1108, 1081 HZ Amsterdam, The Netherlands; 2 Applied Physiology Laboratory, Kobe Design University, Kobe, Japan; 3 Japan Society for Promotion of Sciences, Tokyo, Japan; 4 Osaka International University, Moriguchi, Japan; 5 Graduate School of Health and Sports Science, Doshisha University, Kyoto, Japan; 6 School of Health Sciences, Liverpool Hope University, Liverpool, MSY UK; 7 Applied Physiology Laboratory, Kobe University, Kobe, Japan; 8 Departments of Anatomy and Physiology, and Kinesiology, Kansas State University, Manhattan, KS USA

**Keywords:** Near-infrared spectroscopy, Oxygen delivery, Oxygen utilization, Exercise tolerance, Muscle activation

## Abstract

**Purpose:**

We tested the hypothesis that incremental ramp cycling exercise performed in the supine position (*S*) would be associated with an increased reliance on muscle deoxygenation (deoxy[heme]) in the deep and superficial *vastus lateralis* (VLd and VLs, respectively) and the superficial *rectus femoris* (RFs) when compared to the upright position (*U*).

**Methods:**

11 healthy men completed ramp incremental exercise tests in *S* and *U*. Pulmonary $\dot{V}$O_2_ was measured breath-by-breath; deoxy[heme] was determined via time-resolved near-infrared spectroscopy in the VLd, VLs and RFs.

**Results:**

Supine exercise increased the overall change in deoxy[heme] from baseline to maximal exercise in the VLs (*S*: 38 ± 23 vs. *U*: 26 ± 15 μM, *P* < 0.001) and RFs (*S*: 36 ± 21 vs. *U*: 25 ± 15 μM, *P* < 0.001), but not in the VLd (*S*: 32 ± 23 vs. *U*: 29 ± 26 μM, *P* > 0.05).

**Conclusions:**

The present study supports that the impaired balance between O_2_ delivery and O_2_ utilization observed during supine exercise is a regional phenomenon within superficial muscles. Thus, deep muscle defended its O_2_ delivery/utilization balance against the supine-induced reductions in perfusion pressure. The differential responses of these muscle regions may be explained by a regional heterogeneity of vascular and metabolic control properties, perhaps related to fiber type composition.

## Introduction

Understanding the mechanisms governing the response of oxygen uptake ($\dot{V}$O_2_) to exercise is scientifically important as it is a major determinant of exercise tolerance both in health and disease (Bailey et al. 2009; Burnley et al. 2011; Goulding et al. 2017, 2018a, b, 2019a, b, 2020c; Murgatroyd et al. 2011; Rossiter 2010; Whipp and Ward 1992). In this respect, ramp incremental exercise testing facilitates the determination of multiple parameters of aerobic function in a single visit (Whipp et al. 1981) and has thus been used extensively to investigate the relationships between muscle O_2_ delivery ($\dot{Q}$O_2_), $\dot{V}$O_2_, and fractional O_2_ extraction both at the systemic level (Faulkner et al. 1977) and within the periphery (Boone et al. 2009, 2010; Ferreira et al. 2007b; Murias et al. 2013).

Investigations utilizing near-infrared spectroscopy (NIRS) to noninvasively determine the balance of $\dot{Q}$O_2_-to-$\dot{V}$O_2_ within the microcirculation have revealed an approximately sigmoidal or “double-linear” response of deoxygenation (i.e. deoxygenated hemoglobin + myoglobin; deoxy[heme]) during ramp exercise, leading some to suggest a nonlinear relationship between $\dot{Q}$O_2_ and $\dot{V}$O_2_ within the muscle microcirculation (Boone et al. 2009, 2010; Ferreira et al. 2007b; Spencer et al. 2012). Interventions that perturb the normal balance of $\dot{Q}$O_2_-to-$\dot{V}$O_2_ during incremental exercise have the capacity to further our understanding of this relationship and its role in task failure. In this regard, supine exercise reduces $\dot{Q}$O_2_/$\dot{V}$O_2_ via a loss of perfusion pressure due to the absence of the hydrostatic gradient effect in this position (DiMenna et al. 2010a; Jones et al. 2006; Koga et al. 1999). Accordingly, supine exercise increases the slope of the muscle deoxy[heme] response to ramp exercise when compared to the upright position, at least within the superficial *vastus lateralis* (VLs) muscle (DiMenna et al. 2010a). This behavior was associated with a greater slope of $\dot{V}$O_2_ vs. work rate during the latter portion of the ramp during supine compared to upright exercise, a finding interpreted as the supine posture exerting a greater effect on oxidative metabolism at higher intensities where type II fiber recruitment is expected to be accentuated (DiMenna et al. 2010a). However, that investigation only examined a single, superficial portion of the VL muscle (DiMenna et al. 2010a) using continuous-wave (CW-) NIRS (which is limited to relative changes in [heme] chromophores), and as such the extent to which these findings apply to other regions of the exercising muscle mass remains unknown.

In contrast, both phase-modulation and time-resolved (TR-) NIRS devices measure absolute values of [heme] chromophores and possess the capacity for multisite sampling (Koga et al. 2011). Such measurements have shown deoxy[heme] responses to be spatially and temporally heterogeneous during ramp cycle exercise (Chin et al. 2011; Okushima et al. 2015, 2016, 2020). In particular, the deep muscle appears to possess a greater $\dot{Q}$O_2_/$\dot{V}$O_2_ ratio during exercise when compared to superficial muscle (Koga et al. 2015,2017,2019; Okushima et al. 2015). It has been suggested that differences between deep and superficial muscle may emanate, in part, from higher blood flow (Heinonen et al. 2010; Kalliokoski et al. 2000, 2003) and a greater proportion of more highly oxidative type I fibres in the deep muscle (Johnson et al. 1973; Lexell et al. 1983). Consideration of both between- and within-muscle heterogeneity is, therefore, necessary to gain a more complete understanding of local control of $\dot{Q}$O_2_/$\dot{V}$O_2_ relationships, and thus the events that conspire to bring about task failure during ramp incremental exercise.

Our recent findings of increased amplitude of the deoxy[heme] in the supine position in response to heavy-intensity, constant-work rate exercise in both deep and superficial muscle suggest that the impairment in $\dot{Q}$O_2_/$\dot{V}$O_2_ brought about by the supine intervention is apparent across the exercising muscle mass (Goulding et al. 2020b). However, it remains unknown to what extent these effects differ as a function of exercise intensity and/or fiber type. Assessment of deep versus superficial muscle (i.e., deep VL [VLd] vs. VLs), along with muscles differing in activation patterns (assessed using electromyography; EMG) and $\dot{Q}$O_2_/$\dot{V}$O_2_ (i.e., VLs vs. superficial *rectus femoris* [RFs]) in response to supine ramp exercise will, therefore, provide a more complete picture surrounding the events that precipitate exercise intolerance in this position.

The aim of the present study was, therefore, to examine the influence of posture (i.e., supine vs. upright) on the muscle deoxy[heme], total[heme] (i.e., deoxy- + oxygenated[heme]), and muscle activation (assessed via EMG) responses to ramp exercise and their heterogeneity within and among the locomotor muscles. In accordance with our recent study (Goulding et al. 2020b), we hypothesized that supine exercise would: 1) increase the absolute values and slopes of the deoxy[heme] responses to exercise; and 2) that this profile would be invariant across muscles and exercise intensities. In assessing multiple sites using combined high-precision TR-NIRS and EMG measurements, we sought to gain insight into the physiological mechanisms underpinning earlier task failure during supine ramp exercise.

## Methods

### Participants

Eleven healthy male participants (age: 22 ± 4 years; height 175 ± 7 cm; weight 69 ± 9 kg) volunteered to take part, providing written informed consent. The experiment was approved by the Human Subjects Committee of Kobe Design University and conformed to the Declaration of Helsinki, with the exception of registration in a database. Participants were instructed to avoid alcohol and strenuous exercise 24 h prior to each visit, not to consume caffeine on the same day as a scheduled laboratory visit and to arrive at least 3 h postprandial. Each test was scheduled at the same time of day ± 2 h, with at least 48 h between visits.

### Experimental overview

All tests took place in a temperature-controlled laboratory that was maintained at 25 ± 1 °C and 50 ± 10% humidity. Each participant visited the laboratory on two separate occasions. All exercise tests were conducted using an electronically braked cycle ergometer (75XL-III; Combi, Tokyo, Japan). A custom-built metal frame with an adjustable chair was attached to the back of the ergometer, on which participants lay flat during the supine exercise tests to enable supine cycling. Handles were available to grip during the supine exercise tests to prevent rear movements when forces were applied to the pedals. Distance from the iliac crest to the crankshaft was recorded in the first visit and replicated in the subsequent visit in the second posture. Throughout all exercise tests, cadence was strictly maintained at 60 rpm using an audible metronome. The order of upright and supine exercise tests was counterbalanced. A familiarization session was conducted in the supine position to familiarize participants with the unusual mode of cycling and minimize intraindividual variation in cycling gait throughout the test.

Participants performed ramp incremental tests in the upright and supine positions on separate days. Each test was preceded by 2-min quiet rest on the ergometer and 4-min baseline cycling at 20 W. This was followed by a ramped, linear increase in work rate of 20 W/min (i.e., 1 W increase every 3 s) until the participant could no longer maintain the required cadence despite strong verbal encouragement. Task failure was defined as the point at which cadence dropped below 55 rpm for longer than 5 s.

### Measurements

#### Pulmonary $\dot{V}$O_2_

Pulmonary gas exchange/ventilation were measured breath-by-breath throughout all tests using the same methods previously published in our laboratory (Koga et al. 2019; Okushima et al. 2020). The breath-by-breath gas exchange system (model AE-300S; Minato-Medical, Osaka, Japan) was calibrated according to the manufacturer’s instructions before each test. Participants breathed through a low-resistance mouthpiece containing a hot-wire flowmeter for the measurement of inspiratory and expiratory flows and volumes. Inspired and expired gases were sampled continuously from the mouth, and O_2_ and CO_2_ fractional concentrations were measured by fast-responding paramagnetic and infrared analyzers, respectively. Gas volume and concentration signals were time aligned to account for the time lag between the signals to calculate $\dot{V}$O_2_ on a breath-by-breath basis. Alveolar gas exchange variables were calculated according to the algorithms published by Beaver et al. (1981).

#### Time-resolved near-infrared spectroscopy

Continuous non-invasive measurements of absolute deoxy[heme], oxy[heme], total[heme] (i.e., deoxy[heme] + oxy[heme]) and tissue O_2_ saturation (i.e., oxy[heme]/total[heme] × 100, S_tO2_) in the RFs and VLs were made using two TR-NIRS devices (TRS-20; Hamamatsu Photonics K.K., Hamamatsu, Japan), while a high-power TR-NIRS device (TRS-20D; Hamamatsu Photonics K.K., Hamamatsu, Japan) was used to measure the same variables in the VLd. The optodes for superficial muscles were placed on the distal sites of the VL and RF parallel to the major axis of the thigh. For deep muscle, the interoptode spacing was 6 cm and the optodes were placed on the proximal site of the VL muscle. The measurement principles and algorithms employed by the equipment (Koga et al. 2007, 2011; Ohmae et al. 2014), as well as the specific measurement procedures used in our laboratory (Adami et al. 2015; Fukuoka et al. 2015; Koga et al. 2015, 2017, 2019; Okushima et al. 2015, 2016, 2020) have been reviewed in detail elsewhere. Adipose tissue thickness (ATT) was measured at each muscle site during the first visit using B-mode ultrasound (Logiq 400; GE-Yokogawa Medical Systems, Tokyo, Japan). To quantify the influence of ATT on NIRS signals, we employed the correction factor of Bowen et al. (2013) with separate correction factors used for each muscle (Craig et al. 2017).

#### Surface electromyography

Surface electromyography (EMG) was measured using electrodes (Bluesensor T-00-S; Ambu, Ballerup; Denmark) attached to three separate bipolar EMG sensors connected to a multichannel data acquisition system (MP100; Biopac Systems, Goleta, CA) through an amplifier (Polyam 4; NIHON SANKETU, Osaka, Japan) to estimate muscle activation patterns near the TRS-NIRS optode sites of the VLs and RFs, as previously described (Koga et al. 2019; Okushima et al. 2020). At the beginning of each visit, participants performed three repetitions of maximal voluntary contractions (MVCs) for 7 s each by extending their leg against a strap attached to a chain, which was in turn attached to a force transducer (T.K.K. 1269f, Takei Scientific Instruments Co., Niigata, Japan), amplifier system (T.K.K. 1268, Takei Scientific Instruments Co., Niigata, Japan) and multichannel data acquisition system (see above), that was hooked onto an immovable bar, while seated upright on a chair (i.e., a joint angle of 90°). Participants rested for 3 min before performing each subsequent MVC. The integrated EMG (iEMG) of the individual muscles was normalized to the highest 1 s iEMG value observed during the 7-s contraction which produced the highest MVC observed during that visit (i.e., to ensure a true maximum value for each participant on any given day) and expressed as a percentage of MVC.

### Data analysis

$\dot{V}$ O_2_ peak was defined as the highest 20 s bin-average value recorded throughout the test. The gas exchange threshold (GET) and mean response time (MRT) were determined as previously described (Boone et al. 2008; Goulding et al. 2017). Peak work rate (WR_peak_) was defined as the highest work rate attained prior to task failure. All NIRS variables (i.e., deoxy[heme], total[heme], and *S*_tO2_) were linearly interpolated to obtain one datum for each NIRS variable every second. The baseline value of each NIRS measurement was calculated as the mean value over the final 30 s of baseline cycling (i.e., at 20 W). The absolute value of each NIRS measurement was then calculated every 60 s from baseline to maximal exercise for each participant. The value for each NIRS (and iEMG, see below) variable at each 60 s time-point (i.e., every 20 W) was calculated as a 15 s average centered on the time at which each specific power output was attained. Maximal NIRS variables were also calculated as the average over the final 15 s of exercise (i.e., the final 5 W during the test). Subsequently, NIRS variables determined at specific time points were converted to the power outputs associated with these time points for all further analyses. All NIRS variables were also expressed as a percentage of WR_peak_ to facilitate comparisons at the same relative exercise intensity. The decision to plot each NIRS variable as a function of absolute work rate and %WR_peak_ rather than $\dot{V}$O_2_ and %$\dot{V}$O_2_ peak was made in light of the fact that local $\dot{V}$O_2_ at each muscle site is unknown and the MRT is not constant during ramp incremental exercise (Iannetta et al. 2020; Keir et al. 2015). To account for potential changes in total[heme], and hence blood volume between positions (Goulding et al. 2020a, b), and to aid in the interpretation of crossover interaction effects (see results), the values for each NIRS variable were calculated as a relative change from baseline every 10% WR_peak_ using the same method described above for comparisons at the same absolute work rates. Consistent with previous research from our laboratory (Okushima et al. 2015, 2016), we found that it was not possible to accurately characterize the deoxy[heme] response to ramp exercise using either sigmoidal or double-linear fitting methods in all participants. We, therefore, calculated the magnitude of change in each NIRS variable between each increment in absolute and relative work rate (i.e., every 20 W as Δdeoxy- and Δtotal[heme]/Δwork rate). The raw EMG signals were band-pass filtered (8–500 Hz) and rectified (Labchart Pro v.8.1.6, ADinstruments, Sydney, NSW, Australia). The integrated EMG (iEMG) signals were normalized to the highest 1 s value attained during the MVC trial (see above) and expressed as a percentage (%MVC). The changes in deoxy- and total[heme] between each 10% increment in WR_peak_ from baseline were normalized by the change in iEMG over the same period (i.e., ΔμM/Δ%MVC).

### Statistical analysis

All values were expressed as mean ± SD. Comparisons of $\dot{V}$O_2_ parameters and WR_peak_ were analyzed by paired-samples *t* test. All NIRS and iEMG variables were analyzed separately for each muscle (VLd, VLs, and RFs) by two-way repeated measures ANOVA, with main effects of work rate (i.e., every 20 W from 20 to 180 W and WR_peak_ or every 10% WR_peak_ from 0 to 100% WR_peak_) and posture (upright vs. supine). Where significant interaction effects were found, Holm-Sidak adjusted post-hoc comparisons were used to locate these differences. Cohen’s *d* and partial-eta squared (*η*^2^_p_) were also calculated as measures of effect size. Statistical software (SigmaPlot 13.0, Systat Software, San Jose, CA) was used for all statistical analyses; figures were produced using GraphPad Prism (ver. 7.02, GraphPad Software, San Diego, USA). Significance was declared when *P* < 0.05.

## Results

Supine exercise resulted in a reduced $\dot{V}$O_2_ peak (supine: 46 ± 4, upright: 53 ± 6 mL.kg^−1^.min^−1^, *d* = 1.50, *P* < 0.001), GET (supine: 25 ± 4, upright: 29 ± 5 mL.kg^−1^.min^−1^, *d* = 1.40, *P* < 0.001), and WR_peak_ (supine: 241 ± 33, upright: 293 ± 38 W, *d* = 1.93, *P* < 0.001) when compared to upright exercise. Moreover, the MRT was greater in the supine compared to the upright position (supine: 78 ± 16, upright: 65 ± 20 s, *d* = 0.86, *P* = 0.009).

**Fig. 1:**
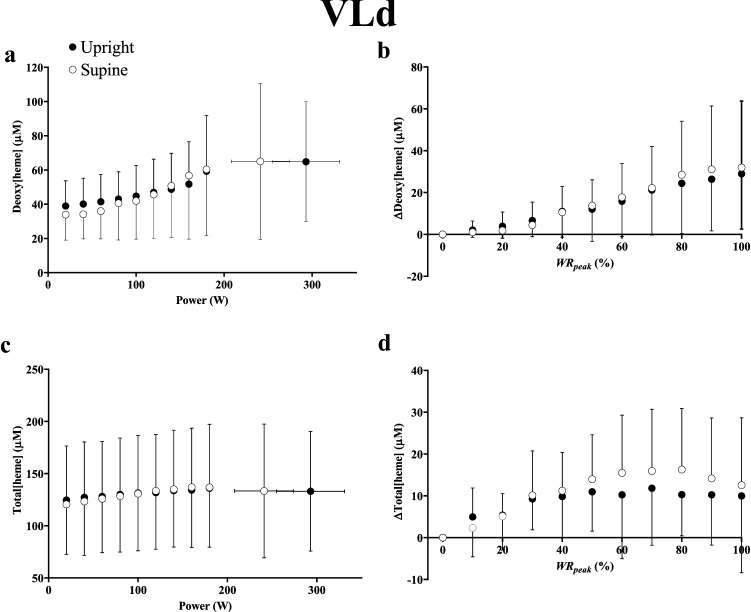
Group mean deoxy[heme] (**a**, **b**) and total[heme] (**c**, **d**) responses to ramp incremental cycle exercise as a function of both absolute power output (**a**, **c**) and as a relative change from the baseline value, plotted as a percentage of the peak work rate attained during the test (**b**, **d**) in the deep *vastus lateralis* (VLd). Error bars represent SD. No differences were observed between postures

**Fig. 2:**
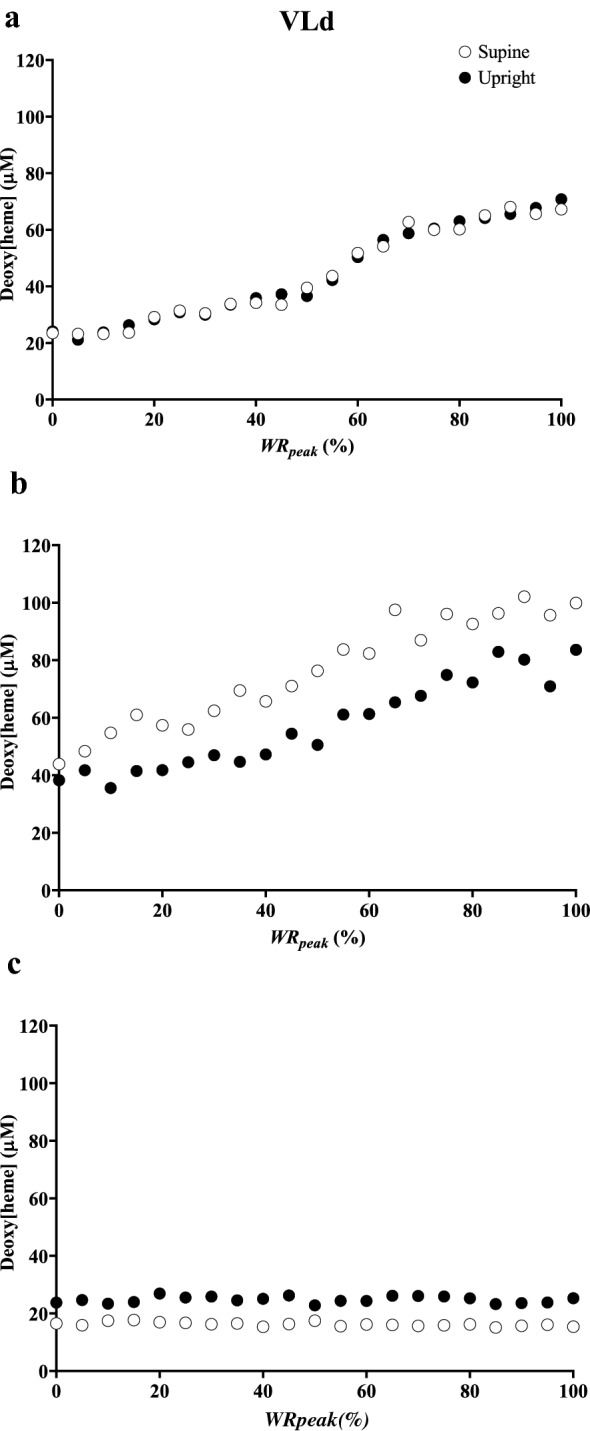
Illustration of the 3 categories of responses of deoxy[heme] to ramp incremental exercise in the upright and supine positions observed in the deep *vastus lateralis* in 3 representative participants. See results section of the text for further details

In the VLd, deoxy[heme] (*η*^2^_p_ = 0.0024, *P* = 0.92) and total[heme] (*η*^2^_p _= 0.0012, *P* = 0.86) did not differ between positions at a given absolute work rate or as a relative change from baseline (*η*^2^_p _= 0.0096–0.022, both *P* > 0.05, Fig. 1). Thus, VLd Δdeoxy[heme]/Δwork rate and Δtotal[heme] /Δwork rate were invariant between positions (*η*^2^_p _= 0.029–0.11, both *P* > 0.05). Consideration of individual responses revealed evidence of interindividual heterogeneity in the ability to defend deoxy[heme] during supine versus upright exercise in the VLd, and representative responses from 3 individuals are illustrated in Fig. 2. In the majority of participants (*n* = 7 out of 11, Fig. 2, Panel A), deoxy[heme] was maintained at concentrations not different from those observed during upright exercise. However, in two participants, there was a greater degree of deoxygenation in the supine versus upright position in the VLd (Fig. 2, Panel b), whereas in the remaining two, no increase in deoxygenation occurred in either position (Fig. 2, Panel c).

**Fig. 3:**
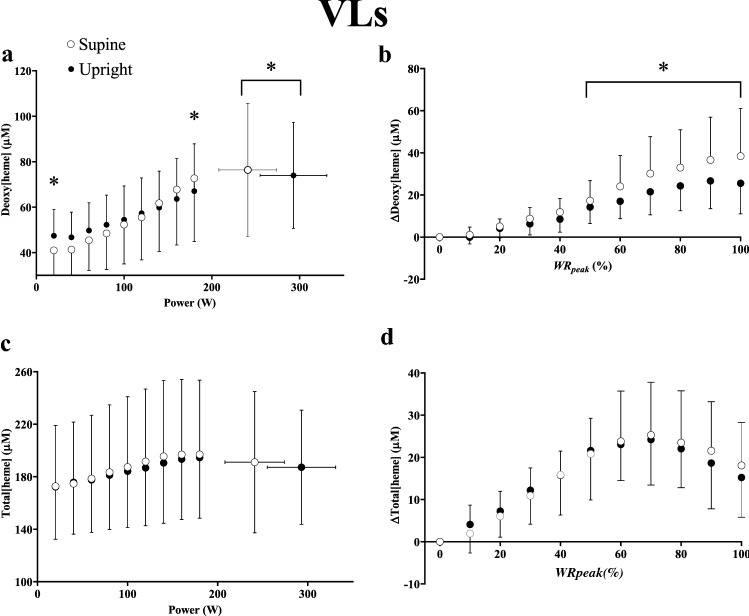
Group mean deoxy[heme] (**a**, **b**) and total[heme] (**c**, **d**) responses to ramp incremental cycle exercise as a function of both absolute power output (**a**, **c**) and as a relative change from the baseline value, plotted as a percentage of the peak work rate attained during the test (**b**, **d**) in the superficial *vastus lateralis* (VLs). Error bars represent SD. *Significant difference between postures at the same absolute or relative work rate (*P* < 0.05)

**Fig. 4:**
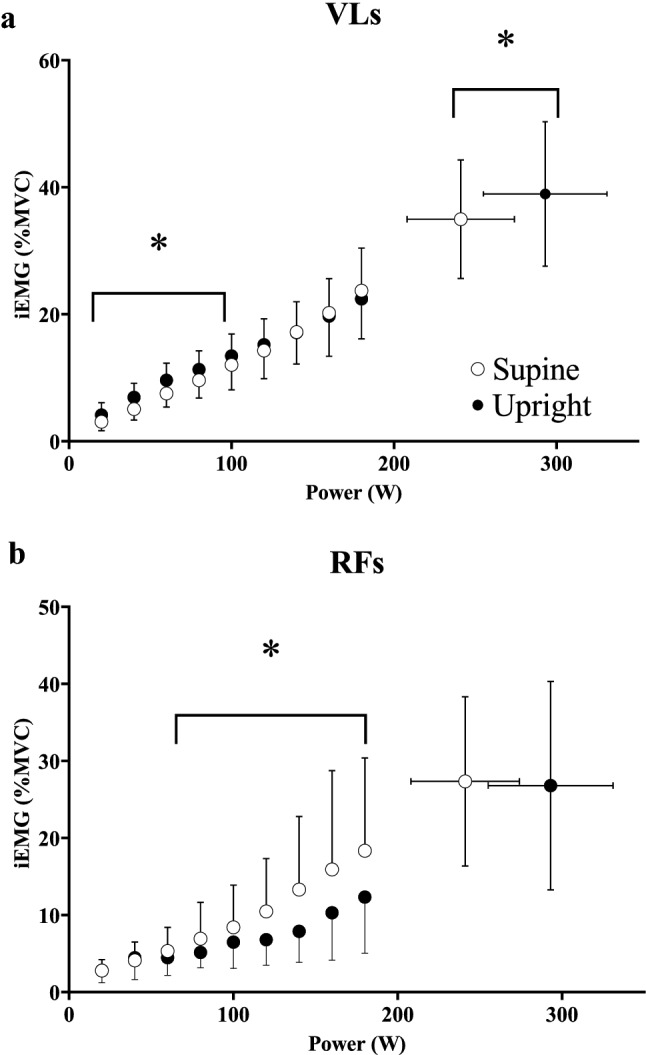
Group mean iEMG profiles in the VLs (**a**) and RFs (**b**) as a function of both absolute power output. Error bars represent SD. *Significant difference between postures at the same absolute or relative work rate (*P* < 0.05)

In the VLs, deoxy[heme] was lower at 20 W but greater at 180 W and maximal exercise in the supine compared to the upright position (i.e., crossover interaction effect, *η*^2^_p _= 0.50, *P* < 0.001, Fig. 3, Panel a). Δdeoxy[heme]/Δwork rate was greater at 120 W, and every work rate thereafter until maximal exercise in the supine position (*η*^2^_p _= 0.27, *P* < 0.001). When expressed as a relative change from baseline, deoxy[heme] was greater from 50 to 100% WR_peak_ in the supine compared to the upright position (*η*^2^_p_ = 0.40, *P* < 0.001, Fig. 3, Panel B). Total[heme] and Δtotal[heme]/Δwork rate did not differ between supine and upright exercise when expressed as a function of either absolute work rate or as a relative change from baseline (*η*^2^_p_ = 0.006–0.01, all *P* > 0.05, Fig. 3, Panels c, d). iEMG was lower in the VLs between 20 and 100 W and at maximal exercise (maximal exercise; supine: 35 ± 9, range 25–55, upright: 39 ± 11, range 16–55% MVC) in the supine position (*η*^2^_p _= 0.24, *P* = 0.016, Fig. 4, Panel a). The overall change in deoxy- (*η*^2^_p_ = 0.35, *P* = 0.019) and total[heme] (*η*^2^_p_ = 0.35, *P* = 0.011) normalized by iEMG (i.e., ΔμM/Δ%MVC at maximal exercise) was greater in the supine position in the VLs.

**Fig. 5:**
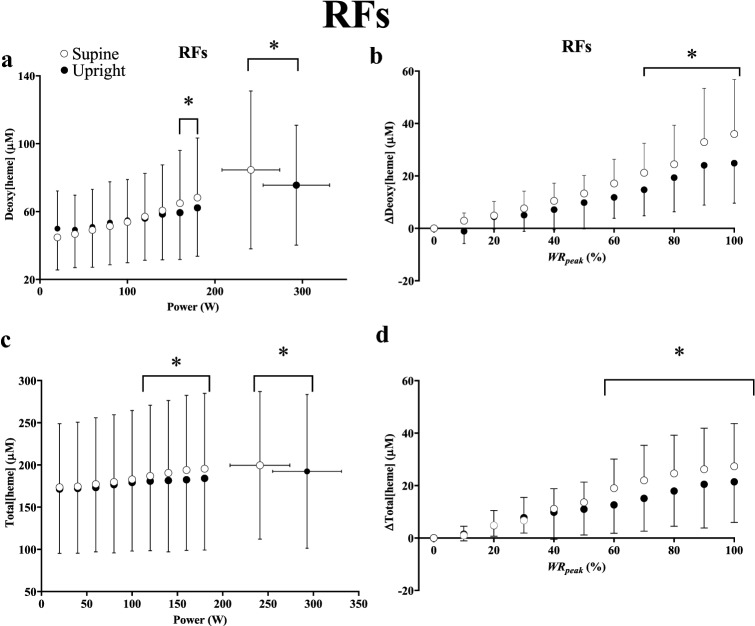
Group mean deoxy[heme] (**a**, **b**) and total[heme] (**c**, **d**) responses to ramp incremental cycle exercise as a function of both absolute power output (**a**, **c**) and as a relative change from the baseline value, plotted as a percentage of the peak work rate attained during the test (**b**, **d**) in the superficial *rectus femoris* (RFs). Error bars represent SD. *Significant difference between postures at the same absolute or relative work rate (*P* < 0.05).

For the RFs, deoxy[heme] was greater at 160 W, 180 W, and maximal exercise (*η*^2^_p_ = 0.27, *P* < 0.001) in the supine position, and when expressed as a relative change from baseline, deoxy[heme] was greater from 70 to 100% WR_peak_ (*η*^2^_p_ = 0.33, P < 0.001, Fig. 5, Panel a, b). Total[heme] was greater in the supine position at 120 W and every work rate thereafter up to maximal exercise (*η*^2^_p_ = 0.38, *P* < 0.001, Fig. 5, Panel c), and when expressed as a relative change from baseline, total[heme] was greater from 60 to 100% WR_peak_ (*η*^2^_p_ = 0.28, *P* < 0.001, Fig. 5, Panel d). Δdeoxy[heme]/Δwork rate and Δtotal[heme]/Δwork rate were greater between each increment in absolute work rate in the supine position (*η*^2^_p_ = 0.014–0.13, both *P* < 0.05). iEMG was greater in the RFs from 80 to 180 W in the supine position (*η*^2^_p_ = 0.24, *P* = 0.016), but not different at maximal exercise (supine: 27 ± 11, range 15–50, upright: 27 ± 14% MVC, range 11– 47, Fig. 4, Panel b). The overall change in deoxy- (*η*^2^_p_ = 0.35, *P* = 0.019) and total[heme] (*η*^2^_p_ = 0.35, *P* = 0.011) normalized by iEMG was greater in the supine position in the RFs.

## Discussion

The principal original finding of the present investigation, consistent with our first hypothesis, was that ramp cycle exercise performed in the supine position resulted in greater absolute values and slopes of the deoxy[heme] responses in the VLs and RFs (i.e., as Δdeoxy[heme]/Δwork rate and as a relative change from baseline) when compared to upright exercise. However, in contrast to our second hypothesis, the effects of the supine posture were highly dependent upon exercise intensity and muscle site. For instance, the effects of supine posture on muscle deoxygenation responses in the superficial muscle (i.e., VLs and RFs) manifested to a greater extent at higher work rates. Moreover, there were no differences between upright and supine exercise in the VLd. That the effects of supine exercise were observed to be specific to muscle region, depth, and exercise intensity might suggest that these differences can be explained by differential effects of impaired perfusion pressure on muscle fiber pools possessing inherently different vascular and metabolic control properties.

### Comparisons with previous work

The present study was designed, in part, to address questions generated by DiMenna et al. (2010a), who demonstrated that the slope of the sigmoid function used to characterize muscle deoxygenation was increased by ~ 100% in the supine position when expressed as a function of absolute work rate and by ~ 70% when normalized to WR_peak_ (DiMenna et al. 2010a). However, that study utilized CW-NIRS, which assumes a fixed scattering coefficient and optical path length throughout data collection. These assumptions are violated during exercise, and as such, CW-NIRS instruments overestimate changes in heme chromophores during ramp exercise (Ferreira et al. 2007a). Ferreira et al. (2007a) also demonstrated that, in some subjects, the change in the reduced scattering coefficient during ramp exercise was related to the increase in total[heme] during the protocol. This latter consideration is particularly pertinent within the context of supine exercise, where we have recently demonstrated that larger changes in total[heme] occur in the transition from rest to exercise compared with the upright position (Goulding et al. 2020a, b). Indeed, in the present study, changes in muscle deoxy[heme] for a given change in work rate between upright and supine exercise in the VLs were more modest than those reported by DiMenna et al. (2010a); with the change in deoxy[heme] for a given change in work rate being increased by ~ 60% in the supine position when expressed in absolute terms and ~ 50% when normalized by WR_peak_. Hence, the present findings highlight the need for caution when interpreting the results of studies that have utilized CW-NIRS to interpret the impact of an intervention on muscle O_2_ extraction.

### Posture-induced changes in superficial muscle

Deoxy[heme] was lower in the VLs during baseline cycling at 20 W during supine compared to upright exercise, a finding likely explained by the lower iEMG (and presumably, therefore, local $\dot{V}$O_2_) in the VLs between 20 and 100 W in the supine position. Above 100 W there were no differences in muscle activation between positions, and as a result Δdeoxy[heme]/Δwork rate was augmented in the supine position above 120 W or 50% WR_peak_ (Fig. 3, Panels a and b). The impact of supine exercise on muscle deoxygenation in the VLs was most evident at the greatest intensities attained: values for deoxy[heme] were only greater in the supine position at 180 W and maximal exercise which occurred despite lower muscle activation at maximal exercise in this muscle (Fig. 4a). Moreover, deoxy[heme] normalized by iEMG (i.e., ΔμM/Δ%MVC) at maximal exercise in the supine position was greater when compared to upright exercise. In the RFs, Δdeoxy[heme]/Δwork rate was greater throughout exercise in the supine compared to the upright position, suggesting that impairments in the $\dot{Q}$O_2_/$\dot{V}$O_2_ ratio necessitated higher fractional O_2_ extractions to support any given change in external work rate in this muscle. Moreover, in the supine position, iEMG in the RFs increased above that for upright exercise at work rates above 80 W. This was followed by increased absolute values of deoxy[heme] from 160 W to maximal exercise. Collectively, therefore, the present findings suggest that the supine posture exerted its greatest effects on superficial muscle (i.e., the RFs and VLs) at higher intensities where type II fiber recruitment would be expected to dominate (Gollnick et al. 1974; Krustrup et al. 2004). These findings are in broad agreement with the earlier work of DiMenna et al. (2010a), and further those findings by highlighting their muscle-region and depth-specificity.

In the RFs, Δtotal[heme]/Δwork rate was increased throughout exercise in the supine position, leading to an elevated total[heme] above 120 W. These observations suggest that a primary mechanism by which the RFs achieved greater fractional O_2_ extraction at greater work rates in the supine position was via elevated diffusive O_2_ conductance consequent to increased microvascular [hematocrit]. Indeed, capillary red blood cell velocity increases more with contractions in less oxidative rat muscles (Dawson et al. 1987) and faster red blood cell velocity is associated with a higher capillary hematocrit (Kindig et al. 2002). We have previously demonstrated that the RFs is more dependent on O_2_ extraction for a given degree of muscle activation when compared to the VLs (Goulding et al. 2020b), consistent with what would be expected in a muscle consisting of a greater proportion of higher-order, less oxidative fibers (Dahmane et al. 2005; Johnson et al. 1973). Therefore, the findings in the RFs herein might reflect that which would be expected from the recruitment of a muscle comprised of a greater proportion of type II muscle fibers that are inherently more sensitive to reductions in muscle perfusion pressure (i.e., closer to their respective O_2_ delivery “tipping points”; DiMenna et al. 2010b; Goulding et al. 2020a, b; Poole et al. 2007, 2008; Poole and Jones 2012), such as that brought about by supine exercise.

### Posture-induced changes in deep muscle

In the VLd, neither deoxy[heme] nor total[heme] differed between positions either at a given absolute work rate or as a relative change from baseline. In addition, Δdeoxy[heme]/Δwork rate and Δtotal[heme]/Δwork rate did not differ between body positions in the VLd. Hence, in the present study and in stark contrast with superficial muscle, deep muscle was not impacted by changes in muscle perfusion pressure induced by the supine position. There are multiple explanations for this finding. For instance, deep muscles receive far greater rates of blood flow during exercise when compared to superficial muscles (Heinonen et al. 2015, 2010; Laughlin and Armstrong 1982; Piiper et al. 1985) and are comprised of a relatively greater portion of oxidative type I fibers (Dahmane et al. 2005; Johnson et al. 1973). These fibers are known to operate at higher $\dot{Q}$O_2_/$\dot{V}$O_2_ ratios and demonstrate less pronounced falls in microvascular *P*O_2_ at the onset of contractions when compared to less oxidative muscle (Behnke et al. 2003; McDonough et al. 2005). Hence, the greater ability of the VLd to defend $\dot{Q}$O_2_/$\dot{V}$O_2_ against changes in perfusion pressure induced by the supine position may be related to this muscle region consisting of a greater proportion of highly oxidative type I fibers with greater rates of blood flow, a higher pressure head for blood-to-myocyte O_2_ flux, and greater vasodilatory control sensitivity (Behnke et al. 2003; Laughlin et al. 2012; McDonough et al. 2005).

The present findings are in contradiction with our recent finding of an increased muscle deoxy[heme] amplitude in the VLd during heavy supine constant work rate exercise (Goulding et al. 2020b). One explanation for this discrepancy may be the fact that muscle deoxy[heme] kinetics are slower during supine compared to upright exercise (Goulding et al. 2020b), and hence the non-steady-state conditions of ramp exercise in the present study may not have allowed sufficient time for steady-state $\dot{Q}$O_2_/$\dot{V}$O_2_ reductions to become manifest in the VLd (Boone et al. 2010). However, in our previous study muscle deoxy[heme] kinetics were also slower in the superficial muscle (VLs and RFs), and in the present study an impaired $\dot{Q}$O_2_/$\dot{V}$O_2_ response was observed during supine ramp exercise in both the RFs and VLs. Hence, an alternative explanation is required.

Individual differences between the two groups of subjects, perhaps in part related to differences in muscle fiber composition, may explain the divergent findings between the present and our previous studies (Goulding et al. 2020a, b). For instance, as shown in Fig. 2, there was evidence of interindividual heterogeneity in the deoxy[heme] responses to exercise in both positions in deep muscle. The factors that enable some subjects to defend $\dot{Q}$O_2_/$\dot{V}$O_2_ during supine exercise in the VLd more effectively than others are presently unclear; for example, the differences in deoxy[heme] between positions were not related to $\dot{V}$O_2_ peak (data not shown). However, it is clear based on the present data that increased fractional O_2_ extraction in the supine position is not an obligatory response in the deep muscle. Hence, at least in the majority of participants, the causes of the earlier task failure in the supine position noted herein (insofar as they involve O_2_ delivery-utilization matching) are primarily confined to superficial muscle.

### Mechanistic bases for reduced exercise tolerance and $\dot{V}$O_2_ peak in the supine position

O_2_ availability is known to modulate muscle recruitment patterns, with lower O_2_ availability driving greater increases in motor unit activity (Moritani et al. 1992, 1993). In the present study, iEMG was lower in the VLs during supine exercise at moderate work rates, suggesting that the reduced perfusion pressure in the supine position may have modulated muscle recruitment patterns. Indeed, iEMG was greater in the RFs during supine exercise above 80 W, implying that lower $\dot{Q}$O_2_/$\dot{V}$O_2_ in the VLs may have necessitated an increased recruitment of the RFs through heavy to severe exercise. Alternatively, the supine posture may have altered muscle recruitment patterns independently of alterations in O_2_ availability, for instance, due to postural changes and/or the loss of the gravitational assist for muscle power production. Irrespective of the mechanism by which muscle recruitment patterns were altered during supine exercise, the muscle deoxygenation profiles observed in the RFs above ~ 160 W are, in turn, consistent with those expected from a muscle comprised of a greater proportion of less oxidative, higher-order motor units (Behnke et al. 2003; Ferreira et al. 2006; Johnson et al. 1973; McDonough et al. 2005), suggesting that exercise in the supine position necessitated the earlier recruitment of more fatigable type II fibers. That Δdeoxy[heme]/ΔiEMG and Δtotal[heme]/ΔiEMG were both increased during supine exercise in the VLs and RFs suggests that supine exercise facilitated the uncoupling of muscle fractional O_2_ extraction and diffusive O_2_ transport from muscle recruitment (Okushima et al. 2020), which would be expected under conditions of altered motor unit recruitment. Hence, the lower recruitment of the VLs at moderate work rates during supine exercise precipitated the earlier recruitment of the RFs, which expresses a phenotype consistent with a muscle composed of a greater proportion of type II fibers when compared to the VLs (Jennekens et al. 1971; Johnson et al. 1973; Okushima et al. 2020). Earlier recruitment of the RFs and a lower $\dot{Q}$O_2_/$\dot{V}$O_2_ in both the RFs and VLs would thus lead to more precipitous drops in microvascular *P*O_2_, intramyocyte *P*O_2_ and the intracellular free energy state as work rate increased (Arthur et al. 1992; Behnke et al. 2003; Hogan et al. 1992; Jackman and Willis, 1996; McDonough et al. 2005; Willis and Jackman, 1994; Wilson et al. 1977), leading to earlier task failure (Grassi et al. 2015; Meyer and Foley 2010).

**Fig. 6:**
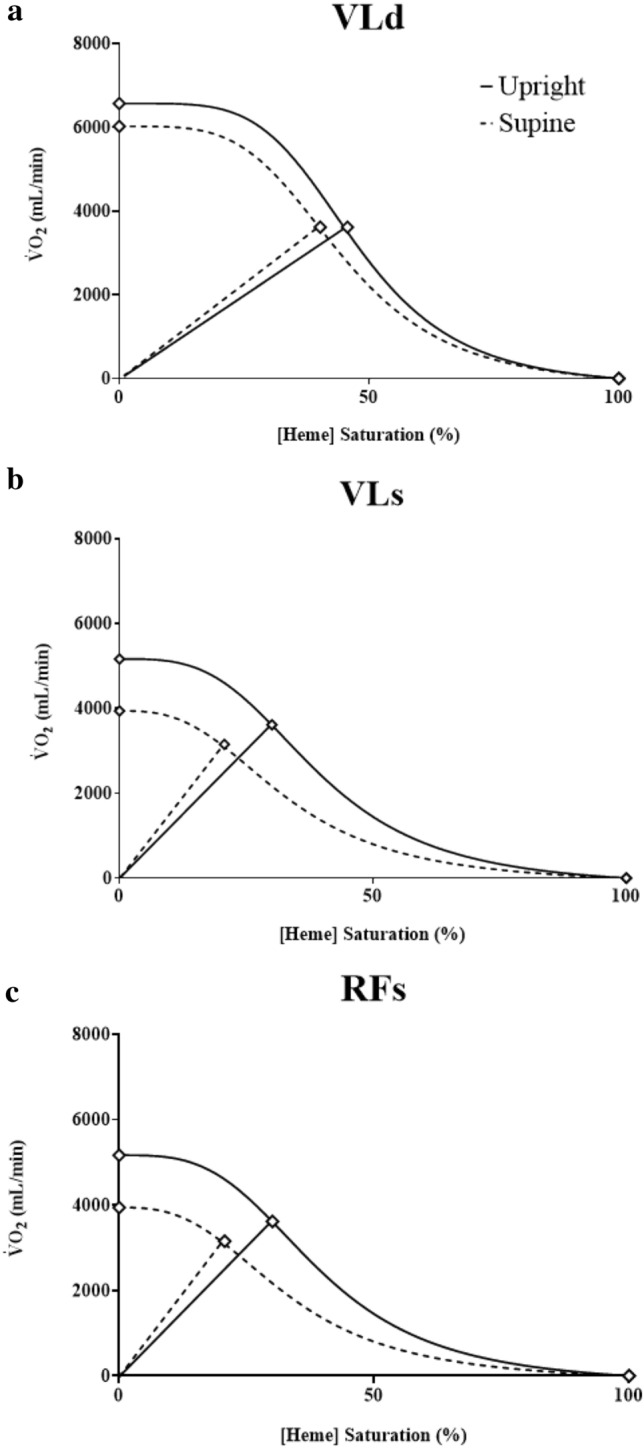
Wagner plots depicting peak regional muscle O_2_ uptake as a function of convective O_2_ delivery (curved lines) vs. tissue O_2_ saturation (assumed to be analogous to mean capillary *P*O_2_ for illustrative purposes) in the VLd (**a**), VLs (**b**), and RFs (**c**). Fick’s law of diffusion depicts $\dot{V}$O_2_ peak as a function of tissue O_2_ saturation with the slope (straight line) representative of muscle O_2_ diffusive conductance. The intersection of these two lines determines the $\dot{V}$O_2_ peak attained. Solid lines represent the upright position, dashed lines represent the supine position

The increased muscle O_2_ extraction at maximal exercise in superficial muscle in the supine position suggests that muscle diffusive O_2_ conductance was slightly enhanced in this position. Despite this, maximal muscle O_2_ extraction was unable to increase sufficiently to offset the reduction in maximal muscle $\dot{Q}$O_2_ and preserve whole-body $\dot{V}$O_2_ peak. Hence, reductions in whole body and regional $\dot{V}$O_2_ peak might be explained, in part, via reductions in convective O_2_ delivery. These concepts are illustrated in Fig. 6, which attempts to explain the contribution of each muscle region studied herein to the reduced whole-body exercise tolerance and $\dot{V}$O_2_ peak (Wagner 1996). Briefly, Fick’s law of diffusion depicts $\dot{V}$O_2_ peak as a function of tissue O_2_ saturation (assumed to be analogous to mean capillary *P*O_2_ for illustrative purposes) with the slope (straight line) representative of muscle O_2_ diffusive conductance. The intersection of these two lines determines the $\dot{V}$O_2_ peak attained. Panel A illustrates that in the VLd, both convective O_2_ delivery and diffusive O_2_ conductance were only marginally impacted by supine exercise, and hence $\dot{V}$O_2_ peak in this muscle is assumed to remain the same. In panels b and c, the increased slope of the Fick diffusion line in the supine position suggests that these superficial muscles rely preferentially on elevated diffusing capacity and O_2_ extraction compared to deeper muscle. However, the increases in diffusive O_2_ conductance were insufficient to offset the reductions in convective O_2_ delivery brought about via supine exercise, hence $\dot{V}$O_2_ peak was reduced.

### Limitations

The depth penetration of NIRS is approximately half of the source-detector separation distance, or around ~ 3 cm for the VLd in the present investigation. As the pathlength estimated in this fashion is a global mean of an infinite number of possible pathlengths travelled by photons on transit from source to detector, this signal likely includes photons returning from the superficial tissues (i.e., VLs). However, that the majority of the signal from the VLd derives from the deeper tissues is borne out by data from validation studies confirming the depth sensitivity of this system using optical phantoms (Koga et al. 2015), and the consistent observation that deep tissue exhibits markedly altered muscle deoxygenation profiles in response to constant work rate (Koga et al. 2015,2017,2019) and ramp exercise (Okushima et al. 2015). Moreover, given the increase in muscle deoxygenation in the VLs in the supine posture noted herein, any contamination of the NIRS signal from deep muscle with more superficial tissue would be expected to weight our data towards producing an effect of the supine posture on muscle deoxygenation in the VLd. Hence, we are confident that the lack of effect of posture on deoxygenation responses in the VLd observed herein reflects the actual responses in the VLd. Another limitation is the fact that neither the degree of muscle activation nor local $\dot{V}$O_2_ were determined in the VLd. Hence, to interpret the dynamic adjustment of $\dot{Q}$O_2_ with respect to a given profile of O_2_ extraction in deep muscle, a $\dot{V}$O_2_ value should be known. However, conducting such measurements in humans during large muscle mass, dynamic exercise is, at present, technically intractable. Further investigations and technological developments (e.g. combined NIRS and diffuse correlation spectroscopy, Quaresima et al. 2019) are, therefore, required to determine how differences in muscle activation and/or local $\dot{V}$O_2_ might contribute to the unique O_2_ transport characteristics of the VLd noted herein.

## Conclusion

In conclusion, marked heterogeneities exist in the strategies by which recruited muscle regions adjust to altered muscle perfusion pressure during supine versus upright ramp incremental cycling exercise. Specifically, deep muscle (i.e., VLd) was able to defend $\dot{Q}$O_2_/$\dot{V}$O_2_ in response to reduced muscle perfusion pressure. In contrast, the perturbation of muscle perfusion pressure in the supine position induced profound changes in $\dot{Q}$O_2_/$\dot{V}$O_2_ and recruitment patterns in superficial muscles. Specifically, the supine position resulted in a proportionally greater recruitment of the RFs and an expedited rate of muscle deoxygenation at higher intensities in both the RFs and VLs when compared to upright exercise. Hence, in the present study, the factors leading to earlier task failure in the supine position were confined to superficial muscle and exerted their greatest effects at higher intensities where type II fiber recruitment would be expected to dominate.

## Data Availability

Data are available upon request from the authors.

## Abbreviations

ATT: Adipose tissue thickness
CO_2_: Carbon dioxide
CW: Continuous-wave
deoxy[heme]: Deoxygenated heme concentration
EMG: Electromyography
iEMG: Integrated electromyography
GET: Gas exchange threshold
MRT: Mean response time
MVC: Maximal voluntary contraction
NIRS: Near-infrared spectroscopy
O_2_: Oxygen
*P*O_2_: Oxygen pressure
$\dot{Q}$O_2_: Rate of oxygen delivery
RFs: Superficial rectus femoris
*S*_tO2_: Tissue oxygen saturation
TR: Time-resolved spectroscopy
VLd: Deep vastus lateralis
VLs: Superficial vastus lateralis
$\dot{V}$O_2_: Rate of oxygen uptake
$\dot{V}$O_2peak_: Highest 20-s rate of oxygen uptake attained during incremental exercise
WR_peak_: Highest work rate value attained during incremental exercise
